# A mobile addiction service for community-based overdose prevention

**DOI:** 10.3389/fpubh.2023.1154813

**Published:** 2023-07-19

**Authors:** Michael D. Pepin, Jillian K. Joseph, Brittany P. Chapman, Christina McAuliffe, Logan K. O’Donnell, Ryan L. Marano, Stephanie P. Carreiro, Erik J. Garcia, Hugh Silk, Kavita M. Babu

**Affiliations:** ^1^UMass Memorial Health, Worcester, MA, United States; ^2^Department of Emergency Medicine, UMass Chan Medical School, Worcester, MA, United States; ^3^Department of Family Medicine and Community Health, UMass Chan Medical School, Worcester, MA, United States

**Keywords:** homelessness, addiction, overdose, community health, suboxone treatment, mobile health, mobile clinic

## Abstract

Mainstays of opioid overdose prevention include medications for opioid use disorder (e.g., methadone or buprenorphine) and naloxone distribution. Inadequate access to buprenorphine limits its uptake, especially in communities of color, and people with opioid use disorders encounter multiple barriers to obtaining necessary medications including insurance, transportation, and consistent availability of telephones. UMass Memorial Medical Center and our community partners sought to alleviate these barriers to treatment through the deployment of a mobile addiction service, called the Road to Care. Using this approach, multidisciplinary and interprofessional providers deliver holistic addiction care by centering our patients’ needs with respect to scheduling, location, and convenience. This program also extends access to buprenorphine and naloxone among people experiencing homelessness. Additional systemic and individualized barriers encountered are identified, as well as potential solutions for future mobile addiction service utilization. Over a two-year period, we have cared for 1,121 individuals who have accessed our mobile addiction service in over 4,567 encounters. We prescribed buprenorphine/naloxone (Suboxone®) to 330 individuals (29.4% of all patients). We have distributed nearly 250 naloxone kits directly on-site or and more than 300 kits via prescriptions to local pharmacies. To date, 74 naloxone rescue attempts have been reported back to us. We have demonstrated that a community-based mobile addiction service, anchored within a major medical center, can provide high-volume and high-quality overdose prevention services that facilitate engagement with additional treatment. Our experience is described as a case study below.

## Introduction

More than 107,000 Americans died from drug overdoses in 2021; the majority of these deaths involved a high-potency opioid (i.e., fentanyl) (1). In 2021, nearly 2,300 people died in Massachusetts alone due to opioid overdoses. In Worcester, Massachusetts, fentanyl is the primary illicit opioid available and is found in 93.3% of opioid-associated deaths (2). Methadone and buprenorphine reduce all-cause and drug-related mortality by nearly 50% (3, 4). When used appropriately, buprenorphine, which does not require daily clinic dispensing, can be an ideal low-barrier starting point on a person’s path to recovery. Unfortunately, access to all medications for opioid use disorder (OUD), particularly buprenorphine, is limited, especially in communities of color (5).

Patients with opioid use disorders (OUD) face multiple individualized and systemic barriers to treatment (6), including long waiting periods for appointments and keeping appointments due to low treatment capacity among healthcare institutions (7), inconsistent access to telephones and transportation (8), and challenges with insurance and personal identification. Prior experiences of stigma in healthcare settings further complicates the engagement of patients with OUD into traditional care paradigms (9).

Opioid use disorders confer a risk of fatal overdose, creating an urgent need to make treatment and harm reduction resources available, yet estimates from 2017 indicate that over 70% of people who needed treatment for OUD did not receive it (10, 11). In other cities, mobile addiction services have created a new care delivery model for deploying lower-barrier treatment, including access to buprenorphine and naloxone. These mobile services meet patients within their contexts and routines, whether that be in a shelter, encampment, or food pantry. Our care delivery was modeled after other successful mobile programs (12), and part of a Massachusetts statewide implementation involving four programs (13).

Mobile addiction services also play an essential role in addressing the intersection of addiction and housing insecurity. Patients experiencing homelessness have higher rates of non-prescription opioid use disorder (16.7% vs. 3.8%) (14), and patients without stable housing are at increased risk of overdose (15). As of January 2020, there were more than 580,000 people in shelters or experiencing street homelessness in the United States (16). The interplay between addiction and housing insecurity is complex. While addictive disorders can contribute to homelessness, the stressors of homelessness can also precipitate or worsen substance use disorder (17). Prior shelter-based addiction treatment efforts with buprenorphine have been effective in promoting sobriety and preventing overdose (18).

## Intervention and implementation

### Concept and initial funding

Prior to the development of the Road to Care, there were brick-and-mortar clinics providing OUD treatment throughout the City of Worcester, with additional harm reduction outreach efforts by different organizations; however, there was no single program that was able to offer accessible clinical and harm reduction services in one place.

UMass Memorial Medical Center (UMMMC), located in Worcester, MA, is the region’s largest safety-net hospital, providing healthcare and health-related services to the uninsured, those insured by Medicaid, and other vulnerable populations regardless of the patient’s ability to pay. The leadership of UMMMC and the Commissioner of the City of Worcester Health and Human Services began meeting in 2019 with a goal of broadening overdose prevention services within our community to align with the anchor mission of UMMMC (19).

Initial explorations of a mobile addiction service made it clear that funding for this type of work would be difficult to support through reimbursements, hospital/health system, or city funding alone. In 2020, Massachusetts Department of Public Health’s Bureau of Substance Addiction Services (MDPH-BSAS) announced a multi-year contract to fund offered $350,000 of operational funding and $50,000 of capital support per year. Additionally, the Kraft Family Foundation donated a mobile clinic for a $0 annual lease to contract awardees as part of the Kraft Center for Community Health’s Community Care in Reach program (10). UMass Memorial Medical Center received an award in the summer of 2020. These funds allowed us to cover salary support for our team to provide a minimum of 24 h of clinical care per week, operational costs, and medical supplies without accessing traditional hospital budgets. Our team began seeing patients in May 2021, carrying medical supplies in backpacks and rolling suitcases to brick-and-mortar shelters and encampments. With the arrival of the vehicle in June 2022, we extended our care to patients in locations throughout the City of Worcester.

Since the contract support both provider and facilities costs, we are able to offer these services without collecting insurance information and at no cost to the patient for care. The majority of our patients are insured, allowing them to obtain medications using their coverage. We are able to support temporary medication costs for patients while they apply for or reinstate their insurance.

In-kind costs are supported by our health system. For our service, securing external funding sources (grants, contracts, philanthropy) to allow no-cost care for our patients has been of critical importance to program implementation and success.

### Determining locations

Prior to starting care delivery, we met with multisectoral city leaders, law enforcement, and community advocacy groups to introduce our planned service and identify concerns about the services provided. Through meetings with the City Manager and his cabinet/ leadership team, we found a supportive environment for the mobile addiction service. We initially selected sites where harm reduction services were in place (e.g., shelters) to ensure a low-friction entry to the community. We subsequently used data-driven processes to select additional sites.

Utilizing a multi-faceted approach, we plan routine or as-needed clinics through identification of areas with a high volume of overdoses. To start, we integrated several sources of data to identify high-need locations. Collaborators, such as emergency medical services (EMS) and community outreach organizations, provided information regarding locations with high incidence of naloxone administration. Additionally, a local university compiled heat maps of the city of Worcester, demonstrating overdose data. City shelters and food pantries represented areas where multiple overdose calls originated. Our collaborations with community organizations that were already providing outreach, the City of Worcester, and local emergency departments provided additional insight into remote encampments and other areas with a high density of overdoses.

Our original sites of care delivery focused on two city shelters, then grew to include a high-volume food pantry, syringe service provider, several encampments, a family-owned local pharmacy, and the city library. We anticipate adding further locations as new, permanent supportive housing systems are introduced later this year. In each of these locations, the owners or administrators of the facility welcomed our team. At each site, we take pains to minimize disruption to pedestrian and business traffic. With the “Road to Care,” we deliberately selected a name that does not specifically identify this service as addiction-focused in order to: (1) be most inclusive for patients that want to see us for other illnesses, and (2) to mitigate friction with neighboring individuals and businesses who may stigmatize addiction services. External signage acknowledges our clinic as part of the Community Care in Reach program while internal signage promotes naloxone and a stigma-free environment in both English and Spanish. The inside of the mobile clinic contains artwork and seasonal decorations to create a warm, welcoming atmosphere for patients.

### Patient care

Patients presenting for care are registered on-site for a same-day appointment. The volume of patients can lead to wait times of up to 30 min. We have increased staffing at our highest volume sites to minimize this delay. A key element of our low-barrier model means that no pre-planning is required on the patient’s part, and there is no penalty or consequence for missed appointments, even if patients register but then leave before being seen. The focus of clinical care is determined by patients. Our providers can manage a breadth of acute and chronic medical and psychiatric conditions. An important aspect of our care is establishing rapport and trust by treating patients’ other health and social needs -- from supplying sleeping bags to addressing foot care.

We offer buprenorphine/naloxone (Suboxone®) for patient-directed induction, offering recommendations for outpatient initiation and micro-dosing. Our team also offers management of stimulant and alcohol use disorders through counseling and medications. Additionally, we make real-time referrals directly to treatment facilities, as well as provide transportation services for individuals requiring more intensive care. At each visit, we promote harm reduction by offering clean injection kits, fentanyl test strips, and naloxone (Narcan®). Our team also offers sexually transmitted infection screening, pre-exposure HIV prophylaxis (PrEP), and condoms.

Our ability to leverage larger health system resources is crucial, particularly for subspecialty care. Once stabilized, patients are referred to brick-and-mortar resources for case management, or office-based psychiatric and addiction treatment. When their acuity of illness cannot be addressed within the resources of the mobile addiction service (e.g., diabetic ketoacidosis, suicidal ideation), patients are transported by ambulance to local emergency departments. The transition of care from the mobile addiction service to the emergency department is often expedited due to the initial evaluation and electronic health record (EHR) documentation conducted by our clinical team in the shared health record.

### Team

The traveling team includes a combination of the physician director, mobile physicians, a mobile physician assistant, recovery specialist, and project manager. Additional team members include financial administrators, research coordinators, as well as consulting providers. The team was selected based on field experience with addictive illness and community engagement. Our team collaborates closely with outreach workers and recovery specialists from multiple Worcester organizations.

The physical and psychological well-being of our team is a top priority, and our street safety plan was derived from other successful outreach organizations. Outreach, whether conducted on the streets or within wooded encampments, requires a minimum of two team members. Given the unique requirements of working within a mobile environment, we provide staff training regarding safe entry and egress from the unit, proper use of restraints while the unit is in motion, fire safety, and fall prevention.

### Community collaborations

Our primary community partner, AIDS Project Worcester, is an essential source of trusted harm reduction services outside of traditional healthcare environments. Offering both brick-and-mortar and outreach care, AIDS Project Worcester is the primary provider of non-medical HIV/AIDS support services in central MA, and has extensive experience with conducting street outreach, sexually transmitted infection screening and treatment, PrEP/PEP, naloxone counseling and distribution, and syringe exchange. Another partner, Eliot Community Human Services is committed to the delivery of high-quality, evidence-based care by creating longstanding relationships with hard-to-reach individuals. The Eliot team has demonstrated expertise in screening for, managing, and referring patients with substance use disorders and concomitant psychiatric disease to treatment. In addition, the Eliot team has a track record of arranging temporary and permanent housing for patients with substance use disorders. In our pilot work, the South Middlesex Opportunity Council (an integrated community-based anti-poverty organization) allowed us to care for patients in their shelter settings. These collaborations now support a larger portfolio of ongoing and planned work.

With these partners, our team has cultivated expertise and experience in caring for patients with opioid use disorders who may also be experiencing homelessness. Through respect for privacy, autonomy, and an emphasis on fostering long-term therapeutic relationships, we have gained the trust of multiple patients living in encampments and have been able to engage them in care. We are continuing to grow our multi-sectoral collaboration to include housing/ case management specialists, law enforcement, harm reduction agencies, and re-entry agencies that support individuals released from incarceration.

Our funders provide a technical assistance meeting each month that allows multiple programs to share challenges and best practices. Additionally, we receive a harm reduction bulletin from MA DPH/BSAS that highlights new and available harm reduction modalities for overdose prevention.

## Evaluation and effects

As part of our agreement with MDPH-BSAS, we collect data and complete an intake form in a system maintained by MDPH-BSAS, which includes demographic information such as gender, ethnicity, race, education, housing status, income, and insurance type (11). We also document encounters as usual within our organization’s EMR and therefore can provide more specific clinical information for data collection. Recognizing the challenges that arise when trying to collect data from people who use drugs and alcohol, the data collection framework is streamlined to emphasize trust, rapport, and clinical relevance (11).

Utilizing a combination of data from the EMR’s demographic and clinical documentation, we report the following metrics to MDPH: number of individuals served; number and nature of service units; number and nature of referrals to medications for opioid use disorder (MOUD); number and nature of referrals to on-going care; number of naloxone rescue kits distributed; number of individuals trained; number of rescue attempts reported; number of syringes distributed, and number of syringes collected; and the number of individuals tested for HIV, HCV, and other infections.

Between May 2021 and April 2023, our team cared for 1,121 individuals who have accessed our mobile addiction service in over 4,567 encounters. Their median age was 43 years (interquartile range 34–55), and 36.5% were female. White patients comprised 60.6% of our population; 10.8% were Black, and 19.9% were Hispanic/Latino. Our patient population is approaching but does not yet mirror the population of greater Worcester which is 13.0% Black and 23.1% Hispanic/ Latino (11). Our low-barrier approach has not yet mitigated known disparities in access to addiction treatment for Brown and Black individuals.

With respect to visit frequency, 48.9% of patients accessed our service only once to date, while 51.1% saw us at least twice. We prescribed buprenorphine/naloxone (Suboxone®) to 330 individuals (29.4% of all patients). Of the patients receiving buprenorphine, 229 (69.4%) saw us more than once, with 130 (39.4%) seeing us at least five times, 66 (20.0%) seeing us at least 10 times, and 22 (6.7%) of patients seeing us at least 20 times. We frequently refer patients in treatment to more traditional treatment settings; these linkages are difficult to measure due to different EHRs and patient privacy.

We have distributed nearly 250 naloxone kits directly on-site or and more than 300 kits via prescriptions to local pharmacies for patients who picking up other prescriptions. To date, 74 naloxone rescue attempts have been reported back to us. Beyond addiction care, our team delivers preventive care, including almost 200 COVID vaccines. Through additional funding, Our most common referral services include psychiatry, general surgery, and dermatology.

We frequently care for wounds related to injection drug use, including non-healing ulcers thought to be related to xylazine use. Our wound care resources range from simple cleansing and debridement to abscess incision and drainage. A point-of-care ultrasound (Butterfly Network; Burlington, MA) has been instrumental in differentiating cellulitis, phlegmon, and abscesses. We believe that the complexity of the wounds seen by our service has increased with the widespread availability of xylazine in our community. Through grant funding, we have been able to create and distribute wound care kits to patients containing gauze, non-adherent dressing material, bandages, antibiotic ointment, gloves, and tape. Through a different grant, we are also able to offer women’s health resources, including contraception and Pap smears.

We are able to access higher levels of care when our resources are exceeded. We have transferred over 20 patients to the ED for a variety of acute processes (e.g., diabetic ketoacidosis, sepsis, soft tissue infections, acute suicidal ideation). During the time that we have been providing care, 16 (1.4%) of our patients died.

## Challenges

Before seeing our first patient, we required a specific EHR (electronic health record) sub-environment nested within our parent hospital’s EHR for this service. Our module allows: (1) immediate, “one-click” registration of patients by program staff; (2) privacy adhering to federal 42 CFR Part 2 to protect individuals with substance use disorders; (3) patient consent for visibility of our records to other providers within our health system for continuity and safety; (4) compliance with standard security protocols despite use of the platform outside of the four walls of the medical center. Expert project managers and analysts guided this work to completion.

Once launched, we faced challenges in reaching individuals with opioid use disorder and concurrent homelessness. Upon encountering this new program, many individuals were reluctant to access our services after experiencing stigma when seeking health care in the past. Critical partnerships with well-known community outreach teams allowed us to meet patients within the context of a trusted relationship. Our volume rapidly grew as we added sites, particularly the high-volume food pantry. Buprenorphine prescriptions initially lagged but have grown as familiarity with our service increased.

Adherence to prescribed buprenorphine was an initial priority; traditionally, non-adherence was believed to leave the patient without the risk reduction inherent in treatment. In contrast, recent data indicate that engagement with treatment reduces overdose mortality, even without adherence to buprenorphine treatment (20). With this in mind, we conduct toxicology testing on a semi-random basis to objectively understand patients’ status in treatment. During initial buprenorphine induction, we clarify initial goals of care with patients (reduced or safer use as opposed to total cessation of use). Providers counsel patients that toxicology screenings are conducted not to assess presence of non-prescription substances, but for the presence of buprenorphine. Most patients are familiar with this expectation and expect that some toxicology testing will be a part of their care plan, given their previous experiences with MAT.

If a patient has serial toxicologic tests (e.g., two or three tests over a period of 2–6 weeks) indicating a lack of buprenorphine adherence, they may be referred to either initiate care with a local methadone provider, offered intensive outpatient program (IOP) referral, or assisted with pursuing inpatient treatment. Our provider team discusses these cases biweekly ensuring a holistic and consistent team-based plan for their care. When referred to other treatment options, these patients are encouraged to continue seeing us for any other health concerns. They are additionally informed that they can restart buprenorphine treatment with us when they feel ready to re-engage with treatment.

We frequently encounter hurdles with inconsistencies in patient access to transportation, leading to missed clinic sessions and challenges obtaining medications from their selected pharmacy. To overcome these challenges, a team clinician was trained in applying for Medicaid-funded transportation to and from clinic sessions. A phone line was also established to allow patients to call a provider directly to address any issues (e.g., need to change pharmacy or refill medications via telehealth due to a missed clinic) in real-time.

One of the most challenging elements of this work occurs after forced removal of encampments by city authorities and law enforcement (“sweeps”). In several cases, we had engaged patients with our service and were amid providing long-term treatment when these individuals were displaced, losing belongings and medications in the process. In our experience, encampment removals increase barriers for these patients and enhance their uncertainties in engaging with outreach groups.

## Conclusion

With the implementation of a mobile addiction service in Worcester, MA, our team has extended access to a major medical center through low-barrier community care. The state funding of this work has been foundational to this effort which would fall outside typical medical center or city budgets. Our use of a low-barrier, no-appointment, free care model has led to the rapid uptake of this service within our community. We continue to refine our harm reduction approaches to align with evidence-based practice and engage patients with this novel care delivery service.

### Take-home points

- Optimal opioid use disorder treatment is convenient and integrated into the routines and natural locations of patients. Addiction care should be offered within a holistic context to address multiple aspects of patients’ health.
- A multidisciplinary team, rooted in emergency medicine and family medicine, offers specialized and parallel skills that facilitate comprehensive care for the target population.
- Development of the mobile team should also take priority, ensuring that the program manager and care providers have the appropriate experience and motivation to care for the intended population.
- Cultivating relationships with other like-minded community organizations within the catchment area is incredibly important to the success of the mobile service. These connections allow the mobile service to reach more patients efficiently and expand the referral network to facilitate timely access.
- Identifying where potential patients are located depends on knowledge of the service area but also can be done by communication with local public safety departments as well as with community organizations and resources.
- Knowledge of existing resources reduces wasted time and efforts, particularly around overcoming hurdles and reducing barriers to care. Cooperative relationships with regional organizations jump starts a newly developed team’s effort and expedites the important elements of trust formation and stigma reduction.

## Data availability statement

The raw data supporting the conclusions of this article will be made available by the authors, without undue reservation.

## Author contributions

MP, BC, JJ, and KB performed the material preparation, data collection, and analysis. MP, JJ, BC, and KB wrote the first draft of the manuscript. All authors contributed to the design, implementation and analysis of this work, commented on previous versions of the manuscript, and read and approved the final manuscript.

## Funding

The work was supported by the MA Department of Public Health/Bureau of Substance Addiction Services, the Kraft Center for Community Health, and the Overdose Prevention Fund at UMassMemorial Health/UMass Chan Medical School. The RIZE Foundation funded the wound care supply project while TD Bank has supported our women’s health effort.

## Conflict of interest

The authors declare that the research was conducted in the absence of any commercial or financial relationships that could be construed as a potential conflict of interest.

## Publisher’s note

All claims expressed in this article are solely those of the authors and do not necessarily represent those of their affiliated organizations, or those of the publisher, the editors and the reviewers. Any product that may be evaluated in this article, or claim that may be made by its manufacturer, is not guaranteed or endorsed by the publisher.
